# Short-Term Changes in Salivary IL-6, TNF-α, and Periodontal Inflammation Following Sleeve Gastrectomy in Patients with Obesity: A Randomized Controlled Pilot Study

**DOI:** 10.3390/biomedicines14081759

**Published:** 2026-08-04

**Authors:** Felicia Gabriela Beresescu, Adriana Monea, Alina Ormenisan, Carmen Tegla, Adina Cosarca, Septimiu Voidazan

**Affiliations:** 1 Faculty of Dental Medicine, George Emil Palade University of Medicine, Pharmacy, Science, and Technology of Targu Mures, 38 Gheorghe Marinescu Str., 540142 Targu Mures, Romania; felicia.beresescu@umfst.ro (F.G.B.);; 2 Mures County Emergency Hospital, 540136 Targu Mures, Romania; 3 Faculty of Medicine, George Emil Palade University of Medicine, Pharmacy, Science, and Technology of Targu Mures, 38 Gheorghe Marinescu Str., 540142 Targu Mures, Romania

**Keywords:** bariatric surgery, interleukin-6, obesity, periodontitis, saliva, sleeve gastrectomy, tumor necrosis factor-alpha

## Abstract

**Background:** Obesity and periodontitis share inflammatory pathways. This pilot randomized controlled trial evaluated salivary IL-6 and TNF-α and periodontal changes after sleeve gastrectomy (SG) versus non-surgical management (NSG). **Methods:** Thirty-two adults with obesity and stage I–III periodontitis were randomized to SG (*n* = 18) or NSG (*n* = 14) and assessed at baseline, 3 months, and 6 months. Primary outcomes were salivary IL-6 and TNF-α; secondary outcomes were PI, BOP, PPD, and CAL. **Results:** At 6 months, SG participants had lower BMI than NSG participants (32.20 ± 3.90 vs. 42.10 ± 4.30 kg/m^2^; *p* = 0.0001). Primary cytokine outcomes also favored SG: IL-6 was 3.72 ± 1.08 vs. 6.34 ± 1.37 pg/mL and TNF-α was 5.11 ± 1.29 vs. 7.71 ± 1.68 pg/mL (both *p* = 0.0001). PI and BOP were lower in SG than NSG (38.60 ± 9.80% vs. 60.80 ± 10.40% and 20.80 ± 7.40% vs. 39.70 ± 9.10%; both *p* = 0.0001), while PPD was modestly lower (2.89 ± 0.43 vs. 3.31 ± 0.46 mm; *p* = 0.017) and CAL was not significantly different (*p* = 0.15). **Conclusions:** These preliminary pilot findings suggest parallel reductions in salivary inflammatory markers and plaque-related/gingival-inflammatory indices after SG, but behavioral confounding, complete-case analysis, short follow-up, and absent significant CAL gain preclude causal or regenerative conclusions.

## 1. Introduction

Obesity is increasingly recognized not only as an excess-adiposity condition but also as a chronic low-grade inflammatory state [1]. Adipose tissue contributes to immune dysregulation through adipokines, macrophage infiltration, oxidative stress, and increased circulating pro-inflammatory cytokines, including IL-6 and TNF-α. These mechanisms are also biologically relevant to periodontal tissue breakdown, where dysregulated host inflammation contributes to connective tissue destruction and alveolar bone loss [2,3].

Periodontitis is classified according to stage and grade under the 2018 World Workshop case definition, integrating severity, complexity, and progression risk [4]. Contemporary clinical guidelines emphasize behavioral risk control, biofilm management, and staged periodontal therapy as core components of care [5]. In patients with obesity, periodontal inflammation may be amplified by systemic inflammation, metabolic impairment, and altered immune responses. Saliva has attracted interest as a non-invasive diagnostic matrix because salivary cytokines can reflect periodontal inflammatory activity; IL-6 and TNF-α are among the mediators most frequently discussed in relation to periodontal disease activity and host response [6].

Oral status and eating habits provide additional clinical links between obesity and periodontal disease. Frequent intake of fermentable carbohydrates, sugar-sweetened beverages, and highly processed foods may increase dental biofilm accumulation and gingival inflammation, whereas periodontal pain, tooth loss, xerostomia, reflux, vomiting episodes, and altered meal frequency after bariatric surgery can modify food choice and oral hygiene practices [5,7,8,9]. The recommended approach for monitoring is therefore multidimensional: periodontal stage/grade diagnosis, repeated full-mouth plaque and bleeding scores, PPD and CAL measurements, medication and oral hygiene documentation, and, in definitive studies, radiographic bone-level assessment [4,5].

Bariatric surgery, including laparoscopic sleeve gastrectomy (SG), produces major weight loss and can reduce systemic inflammatory burden [10]. A meta-analysis of inflammatory markers after bariatric surgery reported reductions in C-reactive protein and IL-6, whereas evidence for TNF-α has been less consistent [11]. More recent longitudinal data suggest that metabolic improvements may precede broader reductions in cytokines, with IL-6 and TNF-α decreasing over follow-up in some bariatric cohorts [12,13].

From a periodontal-methodological perspective, interpretation of postoperative improvement is complex. Plaque-related and gingival inflammatory indices, particularly plaque index and bleeding on probing, are highly responsive to patient behaviour and biofilm control [5], whereas salivary cytokines may reflect both local oral conditions and broader systemic inflammatory status [6,14]. Consequently, observed changes after surgery may arise from enhanced oral hygiene motivation, changes in background medication, reductions in systemic inflammatory burden, or combinations of these factors. Distinguishing among these processes requires careful behavioral and pharmacologic documentation in addition to conventional periodontal assessment.

These issues are especially relevant in early-phase randomized studies. Pilot trials are not designed to establish definitive efficacy, but rather to test feasibility, refine measurement strategies, and identify which clinical and biomarker outcomes are most informative for larger confirmatory studies. In this context, combining salivary IL-6 and TNF-α with periodontal indices provides a clinically meaningful exploratory framework for examining whether metabolic improvement and periodontal inflammatory change occur in parallel after sleeve gastrectomy, even when the causal pathways remain uncertain.

The present pilot randomized controlled study was therefore designed to generate feasibility and preliminary effect-size information for a future definitive trial and to evaluate short-term changes in salivary IL-6 and TNF-α and periodontal clinical parameters in patients with obesity and stage I–III periodontitis undergoing laparoscopic sleeve gastrectomy compared with randomized obese controls receiving non-surgical management over the same intervals.

Clarifying these relationships is clinically relevant because it may help determine whether salivary inflammatory biomarkers can serve as practical adjuncts for monitoring periodontal status in obese patients undergoing metabolic treatment.

## 2. Materials and Methods

### 2.1. Study Design

**Figure 1:**
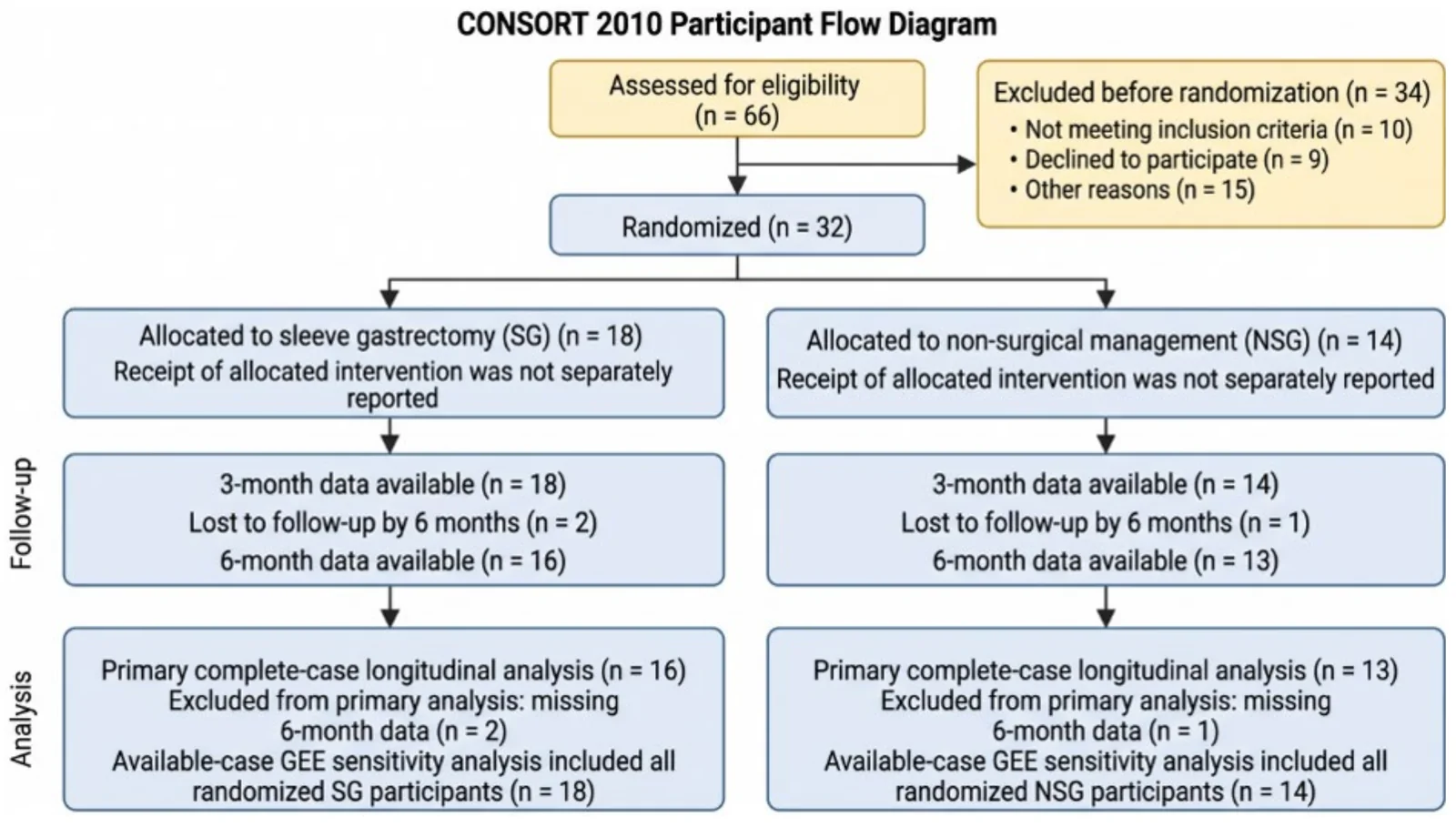
Flowchart Diagram.

This was a randomized, controlled, prospective, longitudinal pilot study with two parallel groups. Patients were recruited from the 2nd Department of Surgery at the Emergency County Clinical Hospital of Targu Mures, Romania, and from associated private practices from December 2022 to April 2025. After eligibility confirmation and completion of baseline periodontal examination and saliva collection, participants were randomly assigned to either the sleeve gastrectomy group (SG) or the non-surgical management group (NSG). Simple computer-generated randomization without blocking or stratification resulted in 18 participants being allocated to SG and 14 participants allocated to NSG. Participants were assessed at baseline (T0), defined as the preoperative visit for SG participants and the enrollment visit for NSG participants, and again at 3 months (T1) and 6 months (T2) (Figure 1).

Randomization and allocation concealment: The random allocation sequence was generated before recruitment using a computer-based random-number procedure by an investigator not involved in periodontal examination or laboratory analysis. Group assignments were placed in sequentially numbered, opaque, sealed envelopes and opened only after baseline measurements had been completed. Due to the nature of bariatric surgery, blinding of participants and treating clinicians was not feasible. The study was conceived as a pilot randomized controlled investigation to assess feasibility, refine measurement procedures, and estimate preliminary effect sizes for a future definitive trial. A formal sample-size calculation for definitive efficacy was not performed. The target sample was based on practical feasibility: the number of eligible bariatric candidates and comparable non-surgical obese participants expected during the recruitment window, surgical scheduling capacity, availability for repeated periodontal and saliva visits, and funding/time limits. The unequal 18/14 allocation arose from simple non-blocked randomization in a small sample; no post-allocation rebalancing was performed because this would have compromised allocation integrity and the pilot objective.

The study protocol was reviewed and approved by the Ethics Committee of the George Emil Palade University of Medicine, Pharmacy, Science, and Technology of Targu Mures, Romania (Approval No. 1863/15 September 2022). Written informed consent was obtained from all participants before enrollment. This investigation was conceived as a randomized controlled pilot study to generate preliminary feasibility and effect-size data for a future definitive trial. The protocol has been registered in the ISRCTN registry under the number ISRCTN70883045.

### 2.2. Study Population

Participants were eligible if they were 18–65 years old and had a BMI ≥ 35 kg/m^2^ with obesity-related comorbidities or a BMI ≥ 40 kg/m^2^. To ensure reliable periodontal examination, participants were required to have at least 12 natural teeth per arch. All participants had stage I–III periodontitis according to the 2018 periodontitis stage/grade classification [4]. Randomization was intended to balance measured and unmeasured baseline characteristics between groups. Baseline comparability was assessed descriptively and statistically for the available demographic, anthropometric, periodontal, and cytokine variables.

Participant-level demographic, medication-screening, background medication, oral hygiene, and periodontal variables were extracted from the available longitudinal dataset. Age was available for 31/32 participants, and sex for 31/32 participants. One NSG participant had missing age and sex fields, and no reason for that missingness was documented in the export available for analysis; this participant was excluded from demographic summaries involving these specific variables. The dataset also contained a plaque-score field that was numerically identical to the full-mouth visible-plaque percentage used as Plaque Index (PI); for clarity and consistency, the manuscript refers to this measure throughout as PI.

Exclusion criteria were smoking more than 10 cigarettes/day, periodontal treatment within the previous 6 months, pregnancy or lactation, previous bariatric surgery, and systemic diseases known to markedly affect periodontal inflammation, such as active autoimmune disorders or malignant disease. Medication-related exclusion criteria applied specifically to recent antibiotics, recent systemic corticosteroids, regular non-steroidal anti-inflammatory drug use within 90 days, and current anti-obesity GLP-1 therapy. In the available data, these screening items were explicitly recorded. Screening was documented from routine eligibility source records using chart review plus participant self-report at enrollment; no separate pharmacy-dispensing linkage or independent medication audit database was available. All enrolled participants passed the baseline medication screen on that basis. Stable background use of antihypertensives, metformin, statins, and proton-pump inhibitors was recorded descriptively, and they were treated as potential confounders rather than exclusionary exposures.

### 2.3. Ethical Considerations

The study protocol was approved by the Institutional Ethics Committee of the participating center and was conducted in accordance with the Declaration of Helsinki. Written informed consent was obtained from all participants before enrollment. The protocol was reviewed and approved by the Ethics Committee of the George Emil Palade University of Medicine, Pharmacy, Science, and Technology of Targu Mures, Romania (Approval No. 1863/15 September 2022).

### 2.4. Anthropometric and Clinical Assessment

Body weight and height were recorded at each visit, and BMI was calculated as weight (kg) divided by height (m) squared. Demographic and clinical data, including age, sex, medical history, obesity-related comorbidities, and current medication use, were recorded from the current dataset. Background medication variables available at each visit included antihypertensive, metformin, statin, and proton-pump inhibitor use. Participant-reported oral hygiene covariates were also available at each visit and included health motivation, brushing frequency, interdental cleaning frequency, mouthrinse frequency, and a composite oral hygiene score. The longitudinal distributions of background medication use, and participant-reported oral-hygiene variables are presented in Supplementary Table S1.

### 2.5. Periodontal Examination and Examiner Calibration

A full-mouth periodontal examination was performed at T0, T1, and T2 by a single calibrated examiner using a manual periodontal probe (UNC-15 or equivalent). Measurements were recorded at six sites per tooth, excluding third molars. The assessed parameters were PI, calculated as the percentage of tooth surfaces with visible plaque and used as a biofilm-control indicator in periodontal monitoring [5]; PPD, measured in millimeters from the gingival margin to the base of the periodontal pocket; CAL, measured in millimeters from the cemento-enamel junction to the base of the pocket; and BOP, recorded dichotomously as present or absent within 15 s after probing. For each participant, full-mouth means that PI, PPD, CAL, and the percentage of BOP-positive sites were calculated.

Examiner calibration was performed before study initiation in 10 patients not included in the final sample. Duplicate measurements were obtained 48 h apart. Intra-examiner reproducibility was assessed using the intraclass correlation coefficient, which exceeded 0.85 for both PPD and CAL.

### 2.6. Standardization of Oral Conditions

All participants received standardized oral hygiene instructions at baseline and were advised to maintain their usual oral care habits throughout follow-up. No periodontal treatment was provided during follow-up unless clinically necessary. Changes in self-care behavior could be described longitudinally because participant-reported oral hygiene variables were available. However, these variables were self-reported and not independently verified by a blinded assessor. They were also not prespecified adjustment covariates in the primary repeated-measures models.

### 2.7. Saliva Collection and Cytokine Analysis

Unstimulated whole saliva samples were collected at each time point between 08:00 and 10:00 a.m. to minimize circadian variability [15]. Participants were instructed to refrain from eating, drinking, smoking, toothbrushing, and mouthrinse use for at least 2 h before collection. Saliva was collected by passive drooling for 5 min into sterile polypropylene tubes, immediately placed on ice, and transported to the laboratory. After centrifugation at 3000 rpm for 10 min at 4 °C, the supernatant was aliquoted and stored at −80 °C until analysis.

Salivary IL-6 and TNF-α concentrations were measured using commercially available ELISA kits according to the manufacturer’s instructions. All samples were analyzed in duplicate, and the mean value was used for statistical analysis. Full assay metadata (for example, manufacturer, catalog number, lot number, detection range, sample dilution, and precision metrics) were retained in the laboratory file, although they were not auditable from the supplied analytical spreadsheet alone.

### 2.8. Outcome Measures

The primary outcomes were longitudinal changes in salivary IL-6 and TNF-α concentrations between baseline and 6 months after surgery or conservative management. Secondary outcomes were longitudinal changes in PI, PPD, CAL, and BOP, as well as exploratory correlations between salivary cytokine concentrations and periodontal clinical parameters. Anthropometric outcomes were treated as contextual outcomes confirming metabolic change after SG.

The periodontal outcomes were interpreted in terms of their biological significance. PI and BOP were considered plaque-related and gingival inflammatory indicators. PPD was interpreted as a clinical pocket measure that may improve through reduced gingival inflammation or reduced inflammatory swelling, especially over short follow-up. CAL was treated as the principal clinical measure of attachment-level change; therefore, the absence of a significant CAL change precludes any conclusion of periodontal attachment regeneration.

### 2.9. Statistical Analysis

Statistical analysis was performed using MedCalc Software, Version 14.5.0.0, supplemented by participant-level analyses from the available dataset for demographic summaries, medication and oral hygiene descriptives, and exploratory correlations. Analyses were performed according to randomized group assignment. Continuous variables were summarized as mean ± standard deviation, and categorical variables as counts and percentages. Baseline between-group comparisons used independent-samples *t* tests, Mann–Whitney tests, Fisher’s exact tests, or chi-square tests as appropriate. Three-time-point within-group analyses used repeated-measures ANOVA with Bonferroni-adjusted pairwise comparisons restricted to strict complete-case observations (participants with observed T0, T1, and T2 values for a given outcome). Because only 29/32 randomized participants had observable 6-month data, a post hoc available-case repeated-measures sensitivity analysis using generalized estimating equations was also performed to examine whether the main directional conclusions were robust under a less restrictive missing-data assumption.

Because all missing longitudinal data occurred at the 6-month visit, the primary repeated-measures ANOVA was restricted to participants with observed T0-T1-T2 data for a given outcome (16 SG and 13 NSG for outcomes available at all visits); no imputation was used in the primary analyses. This preserved a transparent reproduction of the original MedCalc pilot outputs, but it also means that the complete-case estimates may be optimistic if participants with less favorable trajectories were more likely to be lost to follow-up. To assess robustness to this assumption, a post hoc available-case repeated-measures sensitivity analysis was performed using Gaussian generalized estimating equations (GEE) with exchangeable working correlation, allowing participants with observed T0 and T1 values but missing T2 to contribute information. While understandable for this pilot, future definitive trials should prespecify and implement more robust missing data handling techniques, such as multiple imputation or mixed-effects models that can accommodate missing-at-random data more effectively than complete-case analysis, to enhance the reliability of longitudinal estimates. *p* values were interpreted together with effect magnitude, confidence intervals, and clinical plausibility.

Exploratory cytokine-Available periodontal association analyses were performed using Spearman rank correlation coefficients because the study was small and the analyses were exploratory. Correlations were calculated for IL-6 and TNF-α against PI, BOP, PPD, and CAL at T0, T1, and T2, and for T0-to-T2 change scores among participants with complete paired observations. Change scores were defined as T0 minus T2; positive values, therefore, indicate reductions in cytokine or periodontal variables. These analyses were exploratory; *p*-values were not adjusted for multiplicity, and correlations should not be interpreted as causal mediation. Because multiple correlations were tested in a small dataset, the risk of false-positive findings is increased, and any statistically significant correlations should be regarded as hypothesis-generating signals only.

## 3. Results

### 3.1. Participant Flows and Available Data

A total of 32 participants were randomized: 18 to the SG group and 14 to the NSG group. Baseline and 3-month measurements were available for all participants in the dataset. At 6 months, available data were reported for 16 SG participants and 13 NSG participants. Overall attrition at 6 months was 3/32 participants (9.4%), comprising 2/18 participants (11.1%) in the SG group and 1/14 participants (7.1%) in the NSG group. The reason recorded for all missing 6-month observations was loss to follow-up. Because the primary repeated-measures ANOVA required observed T0, T1, and T2 data for a given outcome, these three participants contributed to baseline and 3-month summaries but were excluded from the main longitudinal ANOVA and Bonferroni-adjusted within-group comparisons. Accordingly, complete-case effect estimates should be interpreted cautiously because they may overestimate benefit if attrition favored participants with better trajectories. Follow-up completeness and the reasons recorded for missing 6-month observations are detailed in Supplementary Table S2.

### 3.2. Baseline Comparability

At baseline, the randomized groups were comparable with respect to the available demographic, anthropometric, periodontal, salivary cytokine, background medication, and oral hygiene variables. Age was recorded for all SG participants and for 13 NSG participants. The mean age was 39.83 ± 11.84 years in SG and 45.38 ± 8.70 years in NSG (for the 31 participants where age was recorded; *p* = 0.143). Sex was recorded for all SG participants and for 13 NSG participants; female sex was recorded in 15/18 SG participants and 11/13 NSG participants (*p* = 1.000). Baseline participant-reported oral hygiene measures were also similar between groups, including health motivation, brushing frequency, interdental cleaning frequency, mouthrinse frequency, and the composite oral hygiene score. Baseline demographic, clinical, medication, and oral-hygiene characteristics are summarized in Table 1.

The available dataset contained explicit medication-screening variables. All baseline participants were recorded as screened by chart review plus participant self-report and all passed the medication-exclusion screen; no participant had recent antibiotic exposure, recent systemic corticosteroid use, regular NSAID use within 90 days, or current anti-obesity GLP-1 therapy at enrollment. Stable background medication use was broadly comparable at baseline: antihypertensives were recorded in 8/18 SG participants and 6/14 NSG participants; metformin in 6/18 SG and 4/14 NSG; statins in 3/18 SG and 3/14 NSG; and proton-pump inhibitors in 2/18 SG and 2/14 NSG.

### 3.3. Contextual Anthropometric Outcomes

The SG group experienced substantial weight loss by 3 months and further loss by 6 months. Mean weight decreased from 125.09 ± 11.84 kg at T0 to 105.67 ± 10.66 kg at T1 and 93.40 ± 9.98 kg at T2. Mean BMI decreased from 43.20 ± 4.80 kg/m^2^ to 36.50 ± 4.30 kg/m^2^ at T1 and 32.20 ± 3.90 kg/m^2^ at T2. In complete-case analyses, which were limited to participants with observed data at all three time points (*n* = 16 SG, *n* = 13 NSG), baseline-to-6-month reductions were statistically significant for weight (mean difference 31.597 kg, 95% CI 23.224 to 39.970; *p* < 0.0001) and BMI (mean difference 10.900 kg/m^2^, 95% CI 7.956 to 13.844; *p* < 0.0001). A post hoc sensitivity analysis using available-case data was performed to address potential biases from this complete-case approach.

**Figure 2:**
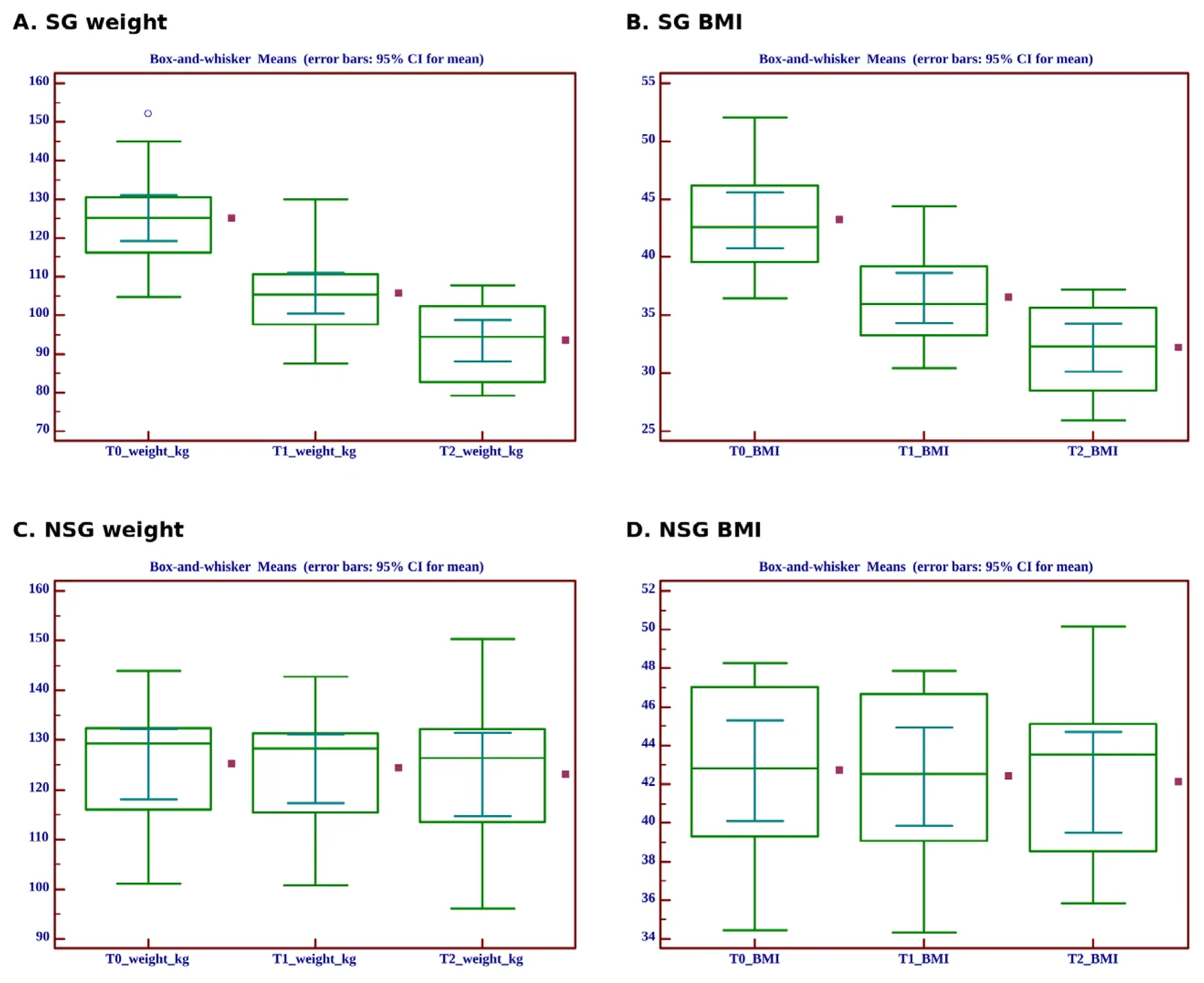
Longitudinal anthropometric trajectories in SG and NSG participants. (**A**) SG weight, (**B**) SG BMI, (**C**) NSG weight, and (**D**) NSG BMI. Boxes show interquartile ranges; lines, medians; squares, means; whiskers, non-outlying ranges; circles, outliers; and error bars, 95% CIs. SG, sleeve gastrectomy; NSG, non-surgical management; T0, baseline; T1, 3 months; T2, 6 months.

In the NSG group, weight and BMI remained broadly stable over 6 months. Although very small T0-to-T1 changes were statistically significant in the supplied repeated-measures output, no baseline-to-6-month changes were significant after Bonferroni correction. Between-group differences were significant at T1 for weight (*p* = 0.0001) and BMI (*p* = 0.001) and remained significant at T2 for both weight and BMI (both *p* = 0.0001) (Figure 2).

### 3.4. Secondary Outcome: Periodontal Clinical Parameters

Periodontal inflammatory variables showed clearer improvement in the SG group compared to the NSG group. In SG participants, PI decreased from 64.30 ± 11.70% at T0 to 49.80 ± 10.50% at T1 and 38.60 ± 9.80% at T2. BOP decreased from 42.60 ± 10.80% to 29.40 ± 8.90% at T1 and 20.80 ± 7.40% at T2. In the complete-case analyses, baseline-to-6-month reductions were statistically significant for PI (mean difference 26.780%, 95% CI 19.444 to 34.116; *p* < 0.0001) and BOP (mean difference 21.169%, 95% CI 15.318 to 27.020; *p* < 0.0001). Given the unblinded nature of the intervention and the significant divergence in self-reported oral hygiene behavior, the observed improvements in plaque-related and gingival-inflammatory findings (PI, BOP) must be interpreted primarily as a consequence of behavioral changes rather than direct evidence of systemic periodontal benefit from surgery. Objectives and blinded assessments of oral hygiene are essential to disentangle these effects in future studies.

The mean PPD in the SG group declined from 3.41 ± 0.52 mm to 2.89 ± 0.43 mm at 6 months. The baseline-to-6-month PPD reduction was statistically significant in complete-case analysis (mean difference 0.536 mm, 95% CI 0.0647 to 1.007; *p* = 0.0236), though the magnitude was clinically modest and, in a short observation period without active periodontal therapy, was most consistent with reduced gingival inflammation or tissue edema rather than true periodontal attachment gain. CAL decreased numerically from 4.08 ± 0.71 mm to 3.63 ± 0.61 mm, but the difference did not remain significant (*p* = 0.2044). Because CAL is the more relevant clinical indicator of attachment-level change, this combined PPD/CAL pattern does not support stable attachment gain, periodontal regeneration, or durable tissue recovery.

**Figure 3:**
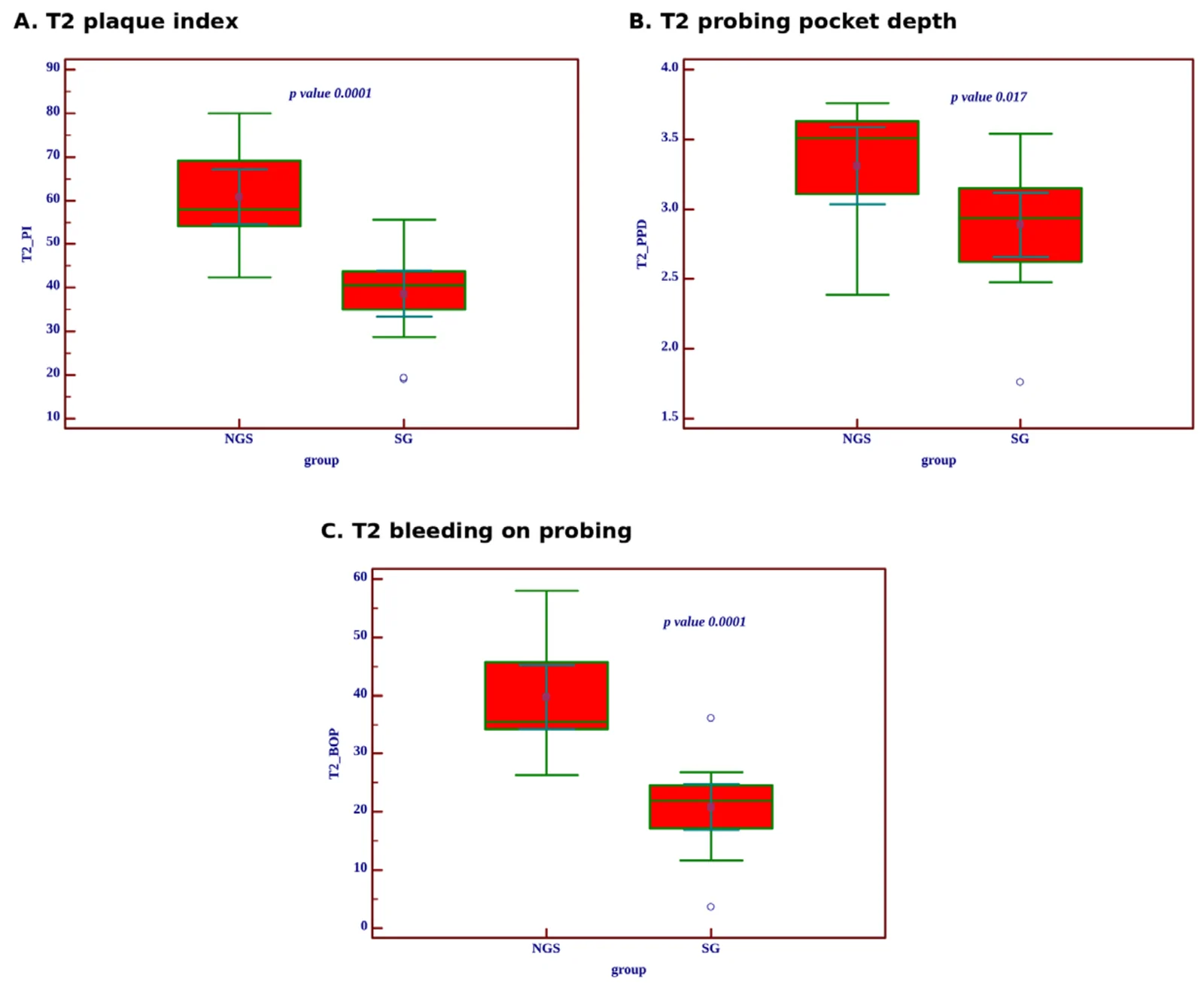
Periodontal outcomes at 6 months in SG and NSG participants. (**A**) PI, (**B**) PPD, and (**C**) BOP in SG and NSG participants. Boxes show interquartile ranges; lines, medians; squares, means; whiskers, non-outlying ranges; circles, outliers; and error bars, 95% CIs. PI, plaque index; PPD, probing pocket depth; BOP, bleeding on probing.

At 6 months, SG participants had statistically lower PI (38.60 ± 9.80% vs. 60.80 ± 10.40%; *p* = 0.0001), BOP (20.80 ± 7.40% vs. 39.70 ± 9.10%; *p* = 0.0001), and PPD (2.89 ± 0.43 mm vs. 3.31 ± 0.46 mm; *p* = 0.017) than NSG participants. However, the PI and BOP differences remain highly susceptible to behavioral confounding because the intervention was unblinded and the SG group showed better self-reported oral hygiene behavior during follow-up. The PPD difference was clinically modest, CAL did not differ significantly between groups at T1 or T2, and the overall periodontal pattern is better interpreted as short-term change in plaque-related and gingival inflammatory status than as evidence of periodontal regeneration or stable attachment gain (Figure 3). Complete cases within-group changes from baseline to 6 months are summarized in Supplementary Table S3.

### 3.5. Primary Outcome: Salivary Cytokine Changes

Salivary IL-6 and TNF-α decreased in the SG group in the primary complete-case analysis. IL-6 declined from 6.84 ± 1.52 pg/mL at T0 to 4.97 ± 1.21 pg/mL at T1 and 3.72 ± 1.08 pg/mL at T2. TNF-α declined from 8.21 ± 1.84 pg/mL at T0 to 6.35 ± 1.46 pg/mL at T1 and 5.11 ± 1.29 pg/mL at T2. In complete-case SG analyses, baseline-to-6-month reductions were statistically significant for IL-6 (mean difference 3.266 pg/mL, 95% CI 2.011 to 4.521; *p* < 0.0001) and TNF-α (mean difference 3.098 pg/mL, 95% CI 1.592 to 4.604; *p* = 0.0002).

In the NSG group, IL-6 and TNF-α changed minimally and showed no significant baseline-to-6-month reduction after Bonferroni correction. Between-group differences favored the SG group at T1 for IL-6 (*p* = 0.002) and TNF-α (*p* = 0.011), and at T2 for both IL-6 and TNF-α (both *p* = 0.0001).

It is important to note that these statistically significant reductions in IL-6 and TNF-α were derived from complete-case analyses, and as discussed in the limitations, may represent optimistic estimates if attrition was not random.

### 3.6. Sensitivity Analysis for Missing Data Handling

**Figure 4:**
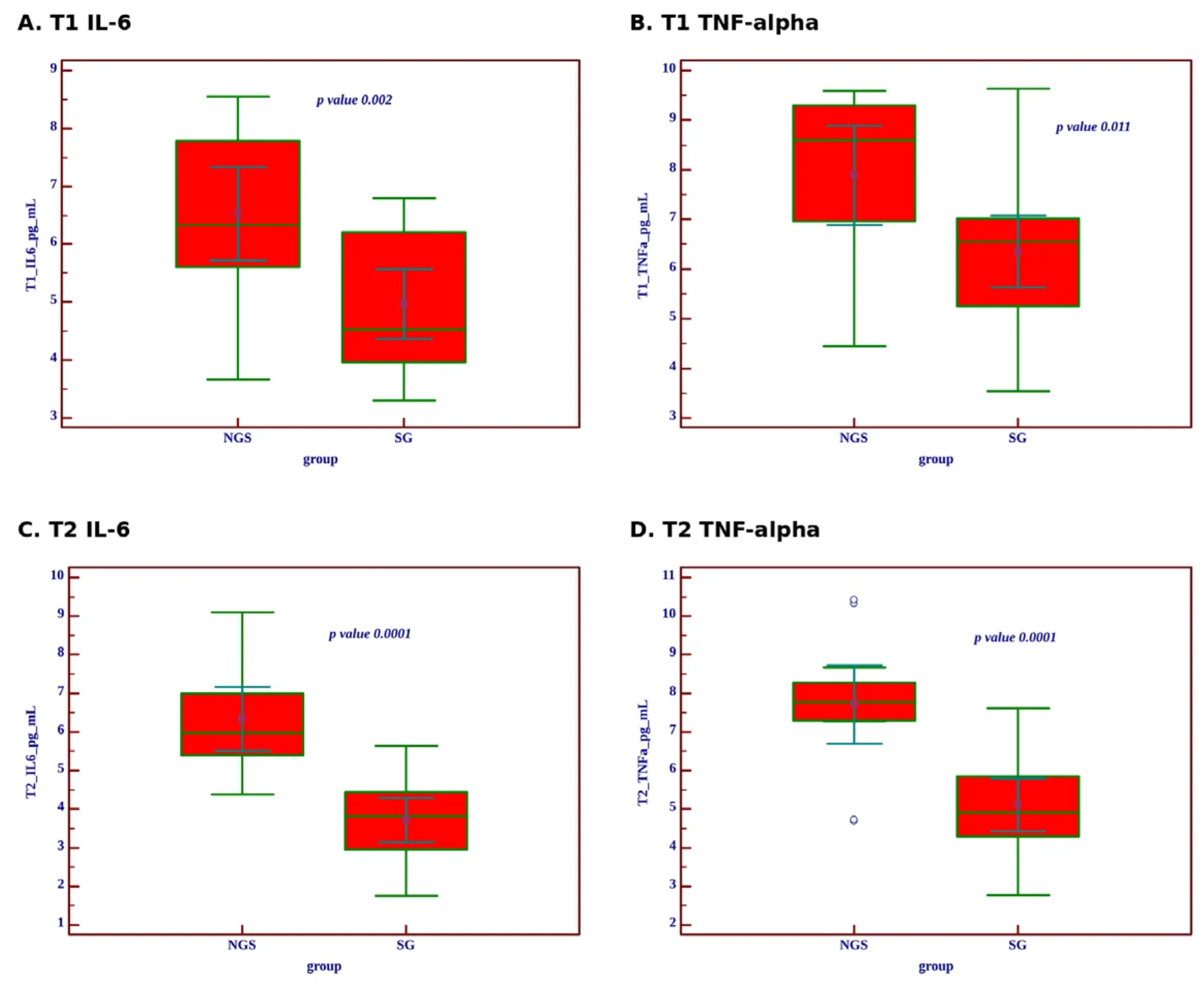
Salivary cytokine outcomes at 3 and 6 months in SG and NSG participants. (**A**) T1 IL-6, (**B**) T1 TNF-α, (**C**) T2 IL-6, and (**D**) T2 TNF-α. Boxes show interquartile ranges; lines, medians; squares, means; whiskers, non-outlying ranges; circles, outliers; and error bars, 95% CIs. T1, 3 months; T2, 6 months.

As a post hoc robustness check, available-case repeated-measures models using Gaussian generalized estimating equations were fitted for BMI, PI, BOP, PPD, CAL, IL-6, and TNF-α so that participants with observed T0 and T1 values but missing T2 could still contribute information. The direction of the primary complete-case findings was unchanged. At T2, SG × time effects remained significant for the primary cytokine outcomes (IL-6, *p* < 0.001; TNF-α, *p* = 0.003), for periodontal outcomes PI (*p* < 0.001), BOP (*p* < 0.001), and PPD (*p* = 0.039), and for BMI (*p* < 0.001), whereas CAL remained non-significant (*p* = 0.238). These results reduce, but do not eliminate, concern that the main conclusions were driven solely by strict complete-case analysis (Figure 4). The descriptive statistics and between-group comparisons at T1 and T2 are summarized in Table 2.

### 3.7. Exploratory Cytokine-Periodontal Correlation Analyses

The planned cytokine-periodontal correlation analyses were performed using paired participant-level observations from the supplied dataset. Spearman’s rank correlations are reported because the study was small and the analyses were exploratory. Cross-sectional correlations were calculated at T0, T1, and T2; change-score correlations were calculated for participants with complete T0 and T2 observations. Because multiple exploratory correlations were examined in a small pilot dataset without multiplicity adjustment, any significant results reported below should be treated as provisional hypothesis-generating signals rather than robust confirmatory findings.

At baseline, most correlations were weak and non-significant, except for a positive TNF-α/BOP signal. By T2, stronger pooled cross-sectional signals were observed between IL-6/TNF-α and PI/BOP. In exploratory change-score analyses, IL-6 reduction correlated with PI and BOP reduction, while TNF-α reduction correlated with BOP reduction. Detailed correlation coefficients and nominal *p*-values are provided in Supplementary Table S4 but should be interpreted with extreme caution as hypothesis-generating signals only, due to the small sample size and lack of multiplicity adjustment.

## 4. Discussion

This randomized, controlled, prospective pilot study suggests that laparoscopic sleeve gastrectomy was associated with 6-month reductions in body weight and BMI, lower salivary IL-6 and TNF-α concentrations, and improvement in several periodontal inflammatory parameters. The primary outcome findings favored SG for both salivary cytokines at 6 months. Secondary periodontal findings favored SG for PI and BOP, and modestly for PPD, while CAL remained statistically similar between groups. These findings should be interpreted as preliminary pilot signals rather than definitive evidence of efficacy or direct biological effects.

Two non-exclusive pathways should be considered. Sleeve gastrectomy may have a direct systemic anti-inflammatory effect through weight loss, reduced adipose-tissue cytokine production, and improved metabolic homeostasis, which could secondarily reduce periodontal inflammatory burden. Surgery and perioperative counseling may indirectly improve oral outcomes by increasing health motivation, self-care, and oral hygiene adherence. The present data cannot definitively distinguish between these mechanisms. However, because PI and BOP are highly responsive to biofilm control and the SG group reported better brushing, interdental cleaning, mouthrinse use, and composite oral hygiene scores during follow-up, the behavioral pathway is the more strongly supported explanation for plaque-related and gingival-inflammatory changes.

The association between salivary IL-6/TNF-α and periodontal inflammation is biologically plausible. In periodontitis, microbial biofilm stimulates epithelial cells, fibroblasts, macrophages, and neutrophils to release cytokines that amplify endothelial activation, leukocyte recruitment, matrix-metalloproteinase activity, RANKL-related osteoclastogenesis, and acute-phase signaling [3,6,16,17]. Obesity may further intensify this oral inflammatory phenotype through adipose-tissue macrophage activation and systemic cytokine spillover [1,2]. Therefore, higher salivary IL-6 and TNF-α may reflect a combined local periodontal burden and systemic metabolic inflammation; nevertheless, saliva integrates multiple sources, so these cytokines should not be interpreted as proof of causal mediation in this pilot dataset.

The periodontal findings themselves should be interpreted with caution and specificity. The most robust periodontal changes were reductions in PI and BOP, both indicators of plaque burden and gingival inflammation, rather than direct evidence of recovered periodontal attachment. Reduced BOP suggests a lower gingival inflammatory burden and is biologically coherent with lower salivary cytokine levels [18,19,20,21]. The observed PPD reduction was statistically significant but modest, and over a 6-month period without active periodontal therapy, it is most likely explained by reduced gingival inflammation and edema rather than true tissue reattachment [21]. Six months is insufficient to determine true periodontal regeneration, sustained attachment gain, or long-term tissue stability [22]. CAL, the more relevant attachment-level change measure, improved numerically but did not reach statistical significance after Bonferroni correction. Accordingly, the study does not demonstrate periodontal regeneration or sustained attachment gain, and the non-significant CAL result should be weighted more heavily than the modest PPD change when interpreting periodontal efficacy.

The literature on bariatric surgery and periodontal/oral outcomes is mixed [19,22,23]. Earlier work reported a high prevalence of periodontitis in both preoperative and postoperative bariatric populations [24], and a 6-month cohort study suggested that bariatric surgery may negatively affect some oral-health outcomes [25]. In contrast, other prospective data indicate that weight loss after bariatric surgery may be associated with decreased periodontal inflammation and improved plaque control [26]. A recent sleeve gastrectomy study in patients with periodontitis reported reductions in plaque and bleeding indices after surgery, while probing depth and clinical attachment loss were largely unchanged, which was a pattern similar to that in the present results [18,27,28,29]. These discrepancies probably reflect differences in sample selection, follow-up duration, oral hygiene protocols, smoking and diabetes status, periodontal case definitions, and whether periodontal treatment was delivered during follow-up.

The cytokine pattern is also consistent with earlier biomarker research. Reports of periodontal status or periodontal therapy have linked salivary IL-6 and TNF-α with active periodontal inflammation [14,30,31,32], while bariatric surgery meta-analytic and longitudinal data generally show reductions in CRP and IL-6 and more variable TNF-α responses after weight loss [11,12,13]. In this study, the SG group showed mean 6-month reductions of 3.266 pg/mL for IL-6 and 3.098 pg/mL for TNF-α, larger than the minimal NSG changes, supporting a biologically plausible oral-systemic inflammatory signal. Absolute concentrations should nevertheless be compared cautiously across studies because saliva collection protocols, ELISA platforms, periodontal severity, diabetes status, medication exposure, and postoperative oral hygiene behaviors differ.

Several mechanisms may explain the observed pattern, but they also highlight possible residual confounding. Reduction in adipose tissue mass after SG may lower the systemic production of pro-inflammatory mediators and alter immune-cell activation. Improvement in metabolic homeostasis may reduce downstream inflammatory signaling [10,33]. At the same time, local biofilm reduction may reduce salivary cytokine release from inflamed periodontal tissues [3,6]. All enrolled participants were documented as screened by chart review plus self-report, and no baseline participant had recent antibiotics, recent corticosteroids, regular NSAID exposure, or current anti-obesity GLP-1 therapy. In addition, baseline background medication use was broadly comparable between groups. Time-varying medication differences did emerge during follow-up, with notably higher proton-pump inhibitor use in SG participants at T1 and lower metformin use in SG participants at T2. As the impact of these medications was not modelled as a time-varying covariate, some of the observed changes in cytokines and clinical outcomes may be due to changes in medication alongside surgery. Future trials should consider incorporating these as time-varying covariates in longitudinal models to better account for their potential influence.

The clinical implication is that bariatric surgery follow-up may be an opportunity to integrate oral health screening and preventive periodontal care [5,9,16]. Patients with severe obesity and periodontitis may benefit from coordinated management involving bariatric, dental, and periodontal teams. The present findings also support including salivary biomarkers as adjunct outcomes in future obesity-periodontitis intervention studies, while recognizing that cytokine concentrations should complement, rather than replace, standardized periodontal clinical measurements.

### Strengths and Limitations

Strengths of this study include a randomized, controlled, prospective design; repeated measurements at clinically meaningful early follow-up intervals; standardized saliva collection procedures; duplicate ELISA measurements; calibrated periodontal assessment by a single examiner; and the availability in the current dataset of explicit medication-screening variables, directly observed background medication use, and participant-reported oral hygiene covariates.

Several limitations materially affect interpretation. Although the available data improved auditability by including medication-screening variables, background medication use, and participant-reported oral hygiene covariates, these factors were not prespecified adjustment covariates in the primary models. Residual confounding therefore remains possible, and self-reported oral hygiene behavior cannot substitute for an objective blinded plaque assessment. These constraints also limit generalizability beyond a small pilot population with stage I–III periodontitis and short-term follow-up.

Because only 29 of 32 randomized participants contributed to the complete-case longitudinal ANOVA, attrition remains an important source of uncertainty. If participants with poorer postoperative trajectories were more likely to be lost, the complete-case estimates could be optimistic. To examine this, we performed a post hoc available-case repeated-measures sensitivity analysis using GEE and all observed repeated measurements. The direction of the primary findings was unchanged: SG × time effects at T2 remained significant for BMI (*p* < 0.001), PI (*p* < 0.001), BOP (*p* < 0.001), PPD (*p* = 0.039), IL-6 (*p* < 0.001), and TNF-α (*p* = 0.003), while CAL remained non-significant (*p* = 0.238). These sensitivity analyses reduce, but do not eliminate, concern that the main conclusions were driven solely by strict complete-case analysis, particularly if missingness was not at random.

The pilot sample size also limits precision. Small samples increase uncertainty around effect estimates, reduce the ability to detect clinically meaningful CAL changes, and make the results vulnerable to the influence of outliers. The modest PPD change therefore should be interpreted alongside the persistently non-significant CAL findings rather than as evidence of stable attachment gain or regeneration. Future definitive trials must prespecify formal sample-size calculations powered for clinically meaningful differences across all primary and key secondary outcomes, with particular emphasis on robust measures of periodontal attachment like CAL, rather than solely relying on plaque-related or short-term inflammatory endpoints.

The cytokine-periodontal correlation analyses were exploratory and unadjusted for multiplicity. Due to the significant separation of the SG and NSG participants at T2, the pooled correlations may be indicative of both group-level differences and individual-level associations. The sample was too small for reliable multivariable adjustment or mediation analysis. Consequently, correlation findings should be viewed as hypothesis-generating signals to guide larger randomized studies rather than as evidence of causal mediation, and the lack of multiplicity adjustment increases the risk of false-positive associations.

Follow-up was limited to 6 months, and no active periodontal therapy was provided during the observation period. Short-term improvements in PI, BOP, and PPD may be transient and may reflect reduced plaque-related inflammation, reduced gingival edema, or soft-tissue changes rather than stable periodontal tissue gain. Six months is insufficient to assess true periodontal regeneration, sustained attachment gain, or long-term tissue stability [21,34,35]. CAL did not improve significantly after Bonferroni correction, and no radiographic bone outcomes were available. To definitively assess periodontal regeneration and long-term stability, future trials must extend follow-up to at least 12 to 24 months and incorporate objective radiographic bone-level assessments, as clinical measures alone (especially PPD without CAL gain) are insufficient over this short timeframe.

Participant oral hygiene practices were measured by self-report and showed clear divergence in favor of the SG group during follow-up. This supports the plausibility of behavioral confounding, but it does not eliminate bias. Self-reported brushing, interdental cleaning, and mouthrinse use are vulnerable to recall error and social-desirability bias, and these variables were not independently verified by a blinded assessor or incorporated as adjustment covariates in the primary repeated-measures models. Future definitive trials should combine blinded professional plaque scoring, standardized behavioral co-interventions, prespecified adherence monitoring, objective oral hygiene endpoints, time-varying covariate adjustment for medication and oral hygiene variables, and longer follow-up with radiographic endpoints to determine whether any apparent periodontal benefit is durable and biologically independent of behavior change.

## 5. Conclusions

Within the limitations of this randomized controlled pilot study, laparoscopic sleeve gastrectomy was associated with significant reductions in salivary IL-6 and TNF-α and short-term improvement in plaque-related and gingival-inflammatory parameters among patients with obesity and stage I–III periodontitis; however, no statistically significant gain in clinical attachment level (CAL) was observed. Nevertheless, the substantial improvements in PI and BOP are likely strongly confounded by concurrent positive changes in oral hygiene behavior reported by participants, and therefore, these findings should not be attributed solely to direct systemic effects of surgery. The modest reduction in PPD, without statistically significant CAL gain, is more indicative of short-term reduced gingival inflammation or edema than stable periodontal attachment recovery or regeneration.

Given the pilot nature, small sample size, and short follow-up, these findings are hypothesis-generating. Future research should involve adequately powered, longer-term trials with formal sample-size calculations focused on attachment-level outcomes, objective blinded oral hygiene assessments, standardized behavioral reinforcement, and prespecified missing-data sensitivity analyses alongside radiographic endpoints. Additionally, prespecifying a limited number of primary correlation hypotheses with appropriate multiplicity adjustments will enable a more robust interpretation of associations between salivary biomarkers and periodontal parameters, clarifying the biological and clinical significance of these results.

## Figures and Tables

**Table 1:** Baseline demographic, clinical, medication-screening, background medication, and self-reported oral hygiene characteristics by group.

Variable	SG *n*	SG Value	NSG *n*	NSG Value	*p* Value
Age (years)	18	39.83 ± 11.84 (20–65)	13	45.38 ± 8.70 (27–58)	0.143
Female sex, *n* (%)	18	15 (83.3%)	13 recorded	11 (84.6%)	1.000
Male sex, *n* (%)	18	3 (16.7%)	13 recorded	2 (15.4%)	1.000
Type 2 diabetes, *n* (%)	18	4 (22.2%)	14	1 (7.1%)	0.355
Hypertension, *n* (%)	18	4 (22.2%)	14	0 (0.0%)	0.113
Dyslipidemia, *n* (%)	18	2 (11.1%)	14	0 (0.0%)	0.492
Screening source: chart + self-report, *n* (%)	18	18/18 (100.0%)	14	14/14 (100.0%)	1.000
Medication screen passed, *n* (%)	18	18/18 (100.0%)	14	14/14 (100.0%)	1.000
Antihypertensive use, *n* (%)	18	8/18 (44.4%)	14	6/14 (42.9%)	1.000
Metformin use, *n* (%)	18	6/18 (33.3%)	14	4/14 (28.6%)	1.000
Statin use, *n* (%)	18	3/18 (16.7%)	14	3/14 (21.4%)	1.000
PPI use, *n* (%)	18	2/18 (11.1%)	14	2/14 (14.3%)	1.000
Health motivation score (1–5)	18	2.44 ± 0.51	14	2.36 ± 0.63	0.678
Toothbrushing frequency/day	18	1.83 ± 0.51	14	2.07 ± 0.27	0.102
Interdental cleaning days/week	18	1.33 ± 1.33	14	1.43 ± 1.45	0.850
Mouthrinse days/week	18	0.94 ± 1.21	14	1.00 ± 1.57	0.914
Composite oral hygiene score (0–10)	18	2.66 ± 1.41	14	2.82 ± 1.47	0.752

Values are mean ± SD or *n* (%). Exploratory *p* values are unadjusted. SG, sleeve gastrectomy group; NSG, non-surgical management group; PPI, proton-pump inhibitor.

**Table 2:** Primary cytokine, secondary periodontal, and contextual anthropometric outcomes at 3 and 6 months.

Variable	SG T1 Mean ± SD	NSG T1 Mean ± SD	T1 *p*	SG T2 Mean ± SD	NSG T2 Mean ± SD	T2 *p*
Weight (kg)	105.67 ± 10.66	124.28 ± 12.03	0.0001	93.40 ± 9.98	123.05 ± 13.88	0.0001
BMI (kg/m^2^)	36.50 ± 4.30	42.40 ± 4.40	0.001	32.20 ± 3.90	42.10 ± 4.30	0.0001
Plaque index (%)	49.80 ± 10.50	61.80 ± 10.60	0.003	38.60 ± 9.80	60.80 ± 10.40	0.0001
BOP-positive sites (%)	29.40 ± 8.90	40.80 ± 9.50	0.002	20.80 ± 7.40	39.70 ± 9.10	0.0001
PPD (mm)	3.12 ± 0.47	3.34 ± 0.48	0.203	2.89 ± 0.43	3.31 ± 0.46	0.017
CAL (mm)	3.89 ± 0.66	4.00 ± 0.68	0.64	3.63 ± 0.61	3.98 ± 0.66	0.15
IL-6 (pg/mL)	4.97 ± 1.21	6.53 ± 1.40	0.002	3.72 ± 1.08	6.34 ± 1.37	0.0001
TNF-α (pg/mL)	6.35 ± 1.46	7.89 ± 1.74	0.011	5.11 ± 1.29	7.71 ± 1.68	0.0001

T1, 3-month follow-up; T2, 6-month follow-up; SG, sleeve gastrectomy group; NSG, non-surgical management group; BMI, body mass index; BOP, bleeding on probing; PPD, probing pocket depth; CAL, clinical attachment level; IL-6, interleukin-6; TNF-α, tumor necrosis factor-alpha; SD, standard deviation. At T1, *n* = 18 in SG and *n* = 14 in NSG. At T2, *n* = 16 in SG and *n* = 13 in NSG.

## Data Availability

The original contributions presented in the study are included in the article, further inquiries can be directed at the corresponding author.

## Supplementary Materials

The following supporting information can be downloaded at: https://www.mdpi.com/article/10.3390/biomedicines14081759/s1, Table S1: Longitudinal background medication and oral-hygiene variables by randomized group; Table S2. Follow-up completeness and reasons for missing 6-month observations; Table S3. Complete case within-group baseline-to-6-month changes; Table S4. Exploratory cytokine-periodontal Spearman correlation analyses using participant-level data.

## Abbreviations

The following abbreviations are used in this manuscript:

ANOVA	Analysis of Variance
BOP	Bleeding on Probing
BMI	Body Mass Index
CAL	Clinical Attachment Level
CRP	C-Reactive Protein
ELISA	Enzyme-Linked Immunosorbent Assay
GEE	Generalized Estimating Equations
GLP-1	Glucagon-Like Peptide-1
HbA1c	Glycated Haemoglobin
IL-6	Interleukin-6
NSAID	Non-Steroidal Anti-Inflammatory Drug
NSG	Non-Surgical Group
PI	Plaque Index
PPD	Probing Pocket Depth
PPI	Proton-Pump Inhibitor
RCT	Randomized Controlled Trial
SD	Standard Deviation
SG	Sleeve Gastrectomy Group
T0	Baseline
T1	3-Month Follow-Up
T2	6-Month Follow-Up
TNF-α	Tumour Necrosis Factor-Alpha
