# Supplementary material for: Short-Term Changes in Salivary IL-6, TNF-α, and Periodontal Inflammation Following Sleeve Gastrectomy in Patients with Obesity: A Randomized Controlled Pilot Study
Source: Biomedicines. 2026 Aug 4;14(8):1759. doi: 10.3390/biomedicines14081759 (PMC13509923; doi:10.3390/biomedicines14081759)
Supplement: Supplementary file 1 [file biomedicines-14-01759-s001.zip › biomedicines-4400994-supplementary.pdf]

Supplementary Table S1. Longitudinal background medication and oral-hygiene variables by randomized group

Variable	SG (T0 / T1 / T2)	NSG (T0 / T1 / T2)	Exploratory p values(T0 / T1 / T2)
Antihypertensive use, n (%)	8/18 (44.4%)6/18 (33.3%)6/16 (37.5%)	6/14 (42.9%)6/14 (42.9%)5/13 (38.5%)	1.0000.7181.000
Metformin use, n (%)	6/18 (33.3%)3/18 (16.7%)0/16 (0.0%)	4/14 (28.6%)4/14 (28.6%)4/13 (30.8%)	1.0000.6690.030
Statin use, n (%)	3/18 (16.7%)4/18 (22.2%)5/16 (31.2%)	3/14 (21.4%)3/14 (21.4%)3/13 (23.1%)	1.0001.0000.697
PPI use, n (%)	2/18 (11.1%)12/18 (66.7%)6/16 (37.5%)	2/14 (14.3%)2/14 (14.3%)2/13 (15.4%)	1.0000.0050.238
Health motivation score (1–5)	2.44 ± 0.513.11 ± 0.583.19 ± 0.66	2.36 ± 0.632.36 ± 0.502.31 ± 0.48	0.6780.0000.000
Toothbrushing frequency/day	1.83 ± 0.512.17 ± 0.382.50 ± 0.63	2.07 ± 0.271.86 ± 0.531.85 ± 0.38	0.1020.0800.002
Interdental cleaning days/week	1.33 ± 1.332.78 ± 1.173.88 ± 1.41	1.43 ± 1.451.07 ± 1.211.62 ± 0.96	0.8500.0000.000
Mouthrinse days/week	0.94 ± 1.212.39 ± 1.383.50 ± 1.51	1.00 ± 1.570.86 ± 1.171.08 ± 1.04	0.9140.0020.000
Composite oral-hygiene score (0–10)	2.66 ± 1.414.30 ± 1.255.35 ± 1.38	2.82 ± 1.472.55 ± 1.262.92 ± 1.04	0.7520.0010.000

Values are listed in T0 / T1 / T2 order. Medication and oral-hygiene variables are directly observed fields from the dataset and are presented as descriptive contextual covariates. Exploratory p values are unadjusted.

Supplementary Table S2. Follow-up completeness and reasons for missing 6-month observations.

Group	Baseline assessed	3-month assessed	6-month assessed	Missing at 6 months	Reason(s) for missing 6-month data
SG	18	18	16	2/18 (11.1%)	Lost to follow-up (n=2)
NSG	14	14	13	1/14 (7.1%)	Lost to follow-up (n=1)
Overall	32	32	29	3/32 (9.4%)	Lost to follow-up (n=3)

All missing observations occurred at the 6-month T2 visit. No missing T0 or T1 visits were recorded in the supplied dataset. Accordingly, no participant was excluded from baseline or 3-month cross-sectional analyses; exclusions occurred only in the three-time-point repeated-measures ANOVA because of missing T2 data.

Supplementary Table S3. Complete case within-group baseline-to-6-month changes.

Variable	SG T0-T2 mean difference (95% CI); Bonferroni *p*	NSG T0-T2 mean difference (95% CI); Bonferroni *p*
Weight (kg)	31.597 (23.224 to 39.970); *p*<0.0001	1.675 (-8.805 to 12.155); p > 0.999
BMI (kg/m^2)	10.900 (7.956 to 13.844); *p*<0.0001	0.656 (-3.041 to 4.354); p > 0.999
Plaque index (%)	26.780 (19.444 to 34.116); p<0.0001	1.604 (-12.172 to 15.379); p > 0.999
BOP-positive sites (%)	21.169 (15.318 to 27.020); p<0.0001	2.251 (-5.776 to 10.279); p > 0.999
PPD (mm)	0.536 (0.0647 to 1.007); p=0.0236	0.0397 (-0.452 to 0.532); p > 0.999
CAL (mm)	0.497 (-0.184 to 1.179); p=0.2044	0.0868 (-0.674 to 0.848); p > 0.999
IL-6 (pg/mL)	3.266 (2.011 to 4.521); p<0.0001	0.312 (-1.256 to 1.881); p > 0.999
TNF-α (pg/mL)	3.098 (1.592 to 4.604); p=0.0002	0.271 (-2.012 to 2.553); p > 0.999

Supplementary Table S4. Exploratory cytokine-periodontal Spearman correlation analyses using participant-level data.

Analysis	Cytokine variable	Periodontal variable	n	Spearman ρ (95% CI)	p value	Finding
T0	IL-6	PI	32	0.25 (-0.11 to 0.55)	0.166	No clear association
T0	IL-6	BOP	32	0.33 (-0.02 to 0.61)	0.067	No clear association
T0	IL-6	PPD	32	0.35 (-0.00 to 0.62)	0.051	No clear association
T0	IL-6	CAL	32	0.32 (-0.04 to 0.60)	0.078	No clear association
T0	TNF-α	PI	32	0.14 (-0.21 to 0.47)	0.429	No clear association
T0	TNF-α	BOP	32	0.36 (0.02 to 0.63)	0.041	Positive signal
T0	TNF-α	PPD	32	0.29 (-0.07 to 0.58)	0.108	No clear association
T0	TNF-α	CAL	32	0.32 (-0.04 to 0.60)	0.078	No clear association
T1	IL-6	PI	32	0.42 (0.08 to 0.67)	0.018	Positive signal
T1	IL-6	BOP	32	0.51 (0.20 to 0.73)	0.003	Positive signal
T1	IL-6	PPD	32	0.35 (0.00 to 0.62)	0.048	Positive signal
T1	IL-6	CAL	32	0.26 (-0.09 to 0.56)	0.146	No clear association
T1	TNF-α	PI	32	0.40 (0.06 to 0.66)	0.024	Positive signal
T1	TNF-α	BOP	32	0.52 (0.21 to 0.73)	0.002	Positive signal
T1	TNF-α	PPD	32	0.37 (0.02 to 0.64)	0.038	Positive signal
T1	TNF-α	CAL	32	0.24 (-0.11 to 0.55)	0.179	No clear association
T2	IL-6	PI	29	0.70 (0.44 to 0.85)	<0.001	Positive signal
T2	IL-6	BOP	29	0.79 (0.59 to 0.90)	<0.001	Positive signal
T2	IL-6	PPD	29	0.51 (0.18 to 0.74)	0.005	Positive signal
T2	IL-6	CAL	29	0.41 (0.06 to 0.68)	0.026	Positive signal
T2	TNF-α	PI	29	0.69 (0.43 to 0.84)	<0.001	Positive signal

p-values are nominal and unadjusted for multiplicity; they should be interpreted as descriptive and hypothesis-generating only.

Δ indicates T0 minus T2 change; positive values indicate reduction/improvement. Spearman rank correlations are shown with approximate 95% confidence intervals. P values are exploratory and unadjusted for multiplicity.

CONSORT 2010 Checklist

Completed page-location form for journal submission

Short-Term Changes in Salivary IL-6, TNF-α, and Periodontal Inflammation following Sleeve Gastrectomy in Patients with Obesity: A Randomized Controlled Pilot Study

Page references correspond to the page numbers printed in the submitted manuscript. “Not reported” and qualifying notes are retained where the manuscript does not fully support the checklist item.

Section / topic	Item	CONSORT 2010 checklist item	Reported on page no.
Title and abstract	**1a**	Identification as a randomised trial in the title.	p. 1 (title)
Title and abstract	**1b**	Structured summary of trial design, methods, results, and conclusions.	p. 1 (Abstract)
Introduction	**2a**	Scientific background and explanation of rationale.	pp. 1–3 (Introduction)
Introduction	**2b**	Specific objectives or hypotheses.	pp. 2–3 (end of Introduction)
Methods — Trial design	**3a**	Description of trial design (for example, parallel or factorial), including the allocation ratio.	p. 3, Section 2.1
Methods — Trial design	**3b**	Important changes to methods after trial commencement, with reasons.	Not reported
Methods — Participants	**4a**	Eligibility criteria for participants.	pp. 3–4, Section 2.2
Methods — Participants	**4b**	Settings and locations where data were collected.	p. 3, Section 2.1
Methods — Interventions	**5**	Interventions for each group, with enough detail to permit replication, including how and when they were administered.	pp. 3–5
Methods — Outcomes	**6a**	Completely defined pre-specified primary and secondary outcomes, including how and when they were assessed.	pp. 5–6, Sections 2.5–2.8
Methods — Outcomes	**6b**	Any changes to trial outcomes after the trial commenced, with reasons.	Not reported
Methods — Sample size	**7a**	How the sample size was determined.	p. 3, Section 2.1
Methods — Sample size	**7b**	When applicable, explanation of interim analyses and stopping guidelines.	Not reported
Methods — Randomisation: sequence generation	**8a**	Method used to generate the random allocation sequence.	p. 3, Section 2.1
Methods — Randomisation: sequence generation	**8b**	Type of randomisation and details of any restriction (such as blocking and block size).	p. 3, Section 2.1
Methods — Allocation concealment	**9**	Mechanism used to implement and conceal the allocation sequence until interventions were assigned.	p. 3, Section 2.1
Methods — Randomisation: implementation	**10**	Who generated the allocation sequence, who enrolled participants, and who assigned participants to interventions.	p. 3, Section 2.1
Methods — Blinding	**11a**	If done, who was blinded after assignment to interventions and how.	p. 3, Section 2.1

Section / topic	Item	CONSORT 2010 checklist item	Reported on page no.
Methods — Blinding	**11b**	If relevant, description of the similarity of interventions.	N/A — surgical and non-surgical interventions were inherently dissimilar
Methods — Statistical methods	**12a**	Statistical methods used to compare groups for primary and secondary outcomes.	p. 6, Section 2.9
Methods — Statistical methods	**12b**	Methods for additional analyses, such as subgroup and adjusted analyses.	p. 6 and pp. 11–12
Results — Participant flow	**13a**	For each group, numbers randomly assigned, receiving intended treatment, and analysed for the primary outcome.	pp. 6–7 and Figure 1, p. 7
Results — Participant flow	**13b**	For each group, losses and exclusions after randomisation, together with reasons.	pp. 6–7, Section 3.1 and Figure 1
Results — Recruitment	**14a**	Dates defining the periods of recruitment and follow-up.	p. 3, Section 2.1
Results — Recruitment	**14b**	Why the trial ended or was stopped.	Not reported
Results — Baseline data	**15**	A table showing baseline demographic and clinical characteristics for each group.	pp. 7–8, Table 1
Results — Numbers analysed	**16**	For each group, the denominator included in each analysis and whether analysis followed original assigned groups.	pp. 6–7 and p. 12
Results — Outcomes and estimation	**17a**	For each primary and secondary outcome, group results plus an effect estimate and precision (such as a 95% confidence interval).	pp. 8–12 and Table 2
Results — Outcomes and estimation	**17b**	For binary outcomes, presentation of both absolute and relative effect sizes.	N/A — principal outcomes were continuous
Results — Ancillary analyses	**18**	Results of other analyses, distinguishing pre-specified from exploratory analyses.	pp. 11–12, Sections 3.6–3.7
Results — Harms	**19**	All important harms or unintended effects in each group.	Not reported
Discussion — Limitations	**20**	Trial limitations, including potential bias, imprecision, and multiplicity where relevant.	pp. 14–16, Strengths and Limitations
Discussion — Generalisability	**21**	Generalisability (external validity and applicability) of the findings.	pp. 14–16
Discussion — Interpretation	**22**	Interpretation consistent with results, balancing benefits and harms and considering other evidence.	pp. 12–16
Other information — Registration	**23**	Registration number and name of the trial registry.	p. 3; non-registration also discussed on p. 14
Other information — Protocol	**24**	Where the full trial protocol can be accessed, if available.	Not reported
Other information — Funding	**25**	Sources of funding and other support, and the role of funders.	p. 16, Funding statement

Notes

This manuscript is a randomized pilot study; the CONSORT 2010 extension for randomized pilot and feasibility trials is also relevant.

The flow diagram supplied with this package retains placeholders for pre-randomization screening/exclusion counts and intervention receipt because those values are not reported in the manuscript.

Reference: Schulz KF, Altman DG, Moher D, for the CONSORT Group. CONSORT 2010
